# A Systematic Review and Implementation Guidelines of Multimodal Foundation Models in Medical Imaging

**DOI:** 10.21203/rs.3.rs-5537908/v1

**Published:** 2025-04-28

**Authors:** Shih-Cheng Huang, Malte Jensen, Serena Yeung-Levy, Matthew P. Lungren, Hoifung Poon, Akshay S Chaudhari

**Affiliations:** Stanford University; Stanford University; Stanford University; Stanford University; Microsoft Research; Stanford University

## Abstract

Artificial Intelligence (AI) holds immense potential to transform healthcare, yet progress is often hindered by the reliance on large labeled datasets and unimodal data. Multimodal Foundation Models (FMs), particularly those leveraging Self-Supervised Learning (SSL) on multimodal data, offer a paradigm shift towards label-efficient, holistic patient modeling. However, the rapid emergence of these complex models has created a fragmented landscape. Here, we provide a systematic review of multimodal FMs for medical imaging applications. Through rigorous screening of 1,144 publications (2012–2024) and in-depth analysis of 48 studies, we establish a unified terminology and comprehensively assess the current state-of-the-art. Our review aggregates current knowledge, critically identifies key limitations and underexplored opportunities, and culminates in actionable guidelines for researchers, clinicians, developers, and policymakers. This work provides a crucial roadmap to navigate and accelerate the responsible development and clinical translation of next-generation multimodal AI in healthcare.

## 1. Introduction

Artificial Intelligence (AI) in healthcare presents significant opportunities to transform clinical workflows and patient care, ultimately improving patient outcomes. Despite numerous attempts to leverage AI models for healthcare, a significant gap remains between AI’s potential and its current usefulness in clinical practice^1–4^. For instance, most contemporary healthcare AI models are constrained by a reliance on single input modalities during training, failing to capture the multimodal nature of medical practice^5^. This contrasts with real-world clinical practice, where physicians rely on diverse data sources to form a holistic view of patient health^6,7^. Moreover, the prevalent use of supervised learning requires extensive, clinical specialist-curated labels, a process that is neither scalable nor cost-effective, leading to models that excel in narrow tasks without broader applicability^8^. Bridging this gap requires a paradigm shift toward AI models that process multimodal inputs and learn from vast, unlabeled datasets, or natural pairs of different modalities, such as medical images and their corresponding reports. These approaches can enhance the performance and usefulness of AI in medical settings, heralding a new era of AI-driven healthcare innovations.

Recently, the AI field has witnessed a leap in capabilities driven by advanced Foundation Models^9^, which possess the attributes necessary for revolutionizing AI in healthcare. Unlike previous generations of specialized models, these Foundation Models can perform a wide variety of tasks using a single model trained on vast amounts of data^9^, typically through a pretraining strategy called Self-Supervised Learning (SSL) (see Terminologies and Strategies for Training Multimodal Foundation Models section). Additionally, these models can exhibit emergent capabilities on tasks for which they were not explicitly trained with^9^. Examples of emergent properties include zero-shot learning, where a model can identify e.g., a disease it was not explicitly trained to classify. While many pioneering Foundation Models are trained with text, which offers a direct semantic interface for humans to intuitively interact with the Foundation Models, these models are not restricted to text only. In fact, several recent research efforts have focused on multimodal Foundation Models that can integrate additional modalities, such as GPT-4V(ision)^10^, LLaVA^11^, and Gemini^12^.

While these models show great promise, they are still in their nascent stages in healthcare. The pathway to developing clinically useful tools remains challenging, demanding improvements in accuracy, safety, and workflow integration. The potential to effect positive changes in healthcare and improve patient outcomes hinges not only on the abilities of model developers but also requires a concerted effort from clinicians, policymakers, and dataset curators^4^. Clinicians play a pivotal role in identifying genuine clinical needs and determining the essential modalities for specific medical tasks. Policymakers are instrumental in updating policies to consider the nuances of multimodal Foundation Models, and striking a balance between streamlining the approval process and keeping a high standard for safety. Dataset curators must prioritize the collection of diverse, representative and multimodal data while maintaining high-quality and clinical relevance. Interdisciplinary collaboration, guided by a shared language and understanding of these complex issues, is crucial to address current challenges and guide future model development.

**Figure 1.**
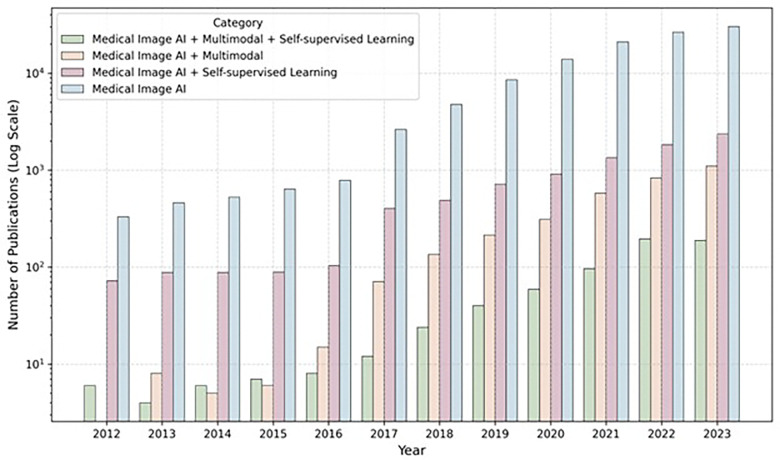
Timeline showing growth in publications on deep learning for medical imaging, based on search criteria applied to PubMed and Scopus. The figure illustrates that multimodal SSL represents a small but rapidly growing subset of medical deep learning literature. Publication counts were aggregated using keyword groups (see Supplementary Table 1). For example, “Medical AI” combines the “Deep Learning” and “Medical Imaging” groups, while “Medical AI + Self-supervised Learning” includes the prior two groups plus the “Self-supervised Learning” group. Specific keywords for each group are detailed in the Methodology section and Supplementary Table 1 and 2. The Y-axis is in log scale.

The objective of this review is threefold: 1) to establish and unify the terminology critical to the intersection of AI and healthcare, with an emphasis on multimodal SSL pretraining (see Terminologies and Strategies); 2) to conduct a systematic review of the emerging field of multimodal Foundation Models for medical imaging applications, extracting key insights and evaluating their current state (see Results); and 3) to highlight the prevailing limitations and actionable future strategies for a broad array of stakeholders, including model developers, clinicians, policymakers, and dataset curators (See Discussion and Guidelines). We focused on multimodality involving medical images, such as radiology images and pathology slides, since medical imaging is an essential part of the diagnostic and treatment workflow across various medical specialties. Although recent trends in medical imaging AI literature increasingly focus on utilizing multimodal Foundation Models (see Fig. 1), with a handful of narrative reviews available^13–16^, there are currently no systematic reviews. By adhering to the PRISMA^17^ guidelines, we methodically gather and consolidate the latest contributions of multimodal Foundation Models for medical imaging applications, providing a comprehensive snapshot of the existing landscape. In total, we screened 1,144 papers and extracted data from 48 papers for this systematic review. Our investigation identifies both challenges and potential solutions for the development of multimodal Foundation Models, with a focus on advancing their usefulness in healthcare.

## 2. Results

**Figure 2.**
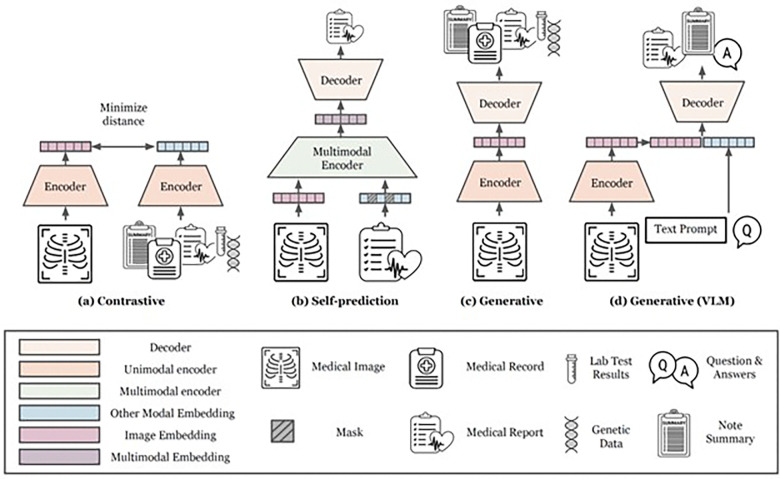
Illustration of multimodal SSL pretraining strategies. During the SSL pretraining stage of multimodal Foundation Models, one or more of the following self-supervised strategies are typically used: (**a**) Contrastive Learning forms positive pairs between matching data with shared semantic content, e.g., X-ray images and reports for the same medical examination, and minimizes the representational distance in a common latent space of positive pairs (**b**) Self-prediction masks out random parts of the inputs and seeks to reconstruct the masked out regions by utilizing complimentary information across the input modalities (**c**) Generative SSL learns the distribution of the training data by generating one or several modalities from another, e.g., generating a report from an X-ray or vice versa (**d**) Generative VLM is a special case of Generative SSL, where an input instruction (“prompt”) can be used to steer the output generated by the model.

**Figure 3.**
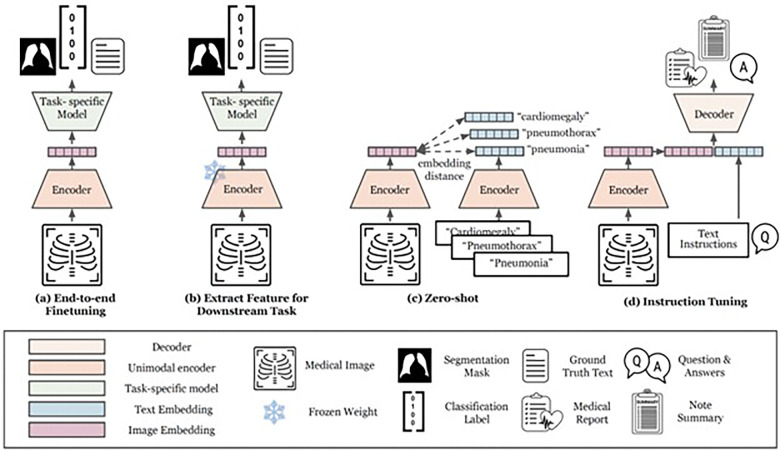
Strategies for adapting SSL pretrained models to downstream tasks. During the fine-tuning stage of a pretrained multimodal Foundation Model, one or several of the following strategies can be used to adapt the model to a given downstream task: (**a**) The entire or parts of the pretrained model are fine-tuned for the downstream task via supervised learning (**b**) The encoder is frozen and used only as a feature extractor, while a task-specific model is trained to utilize these features for the downstream task using supervised learning. (**c**) The pretrained model embeds both the image and text prompts that describe potential classes, and subsequently assigns the class whose text embedding is closest to the image embedding in the shared latent space, enabling zero-shot classification without additional training. (**d**) The model, typically a VLM, is fine-tuned using pairs of instructions and expected outputs for the downstream task (instruction tuning).

**Figure 4.**
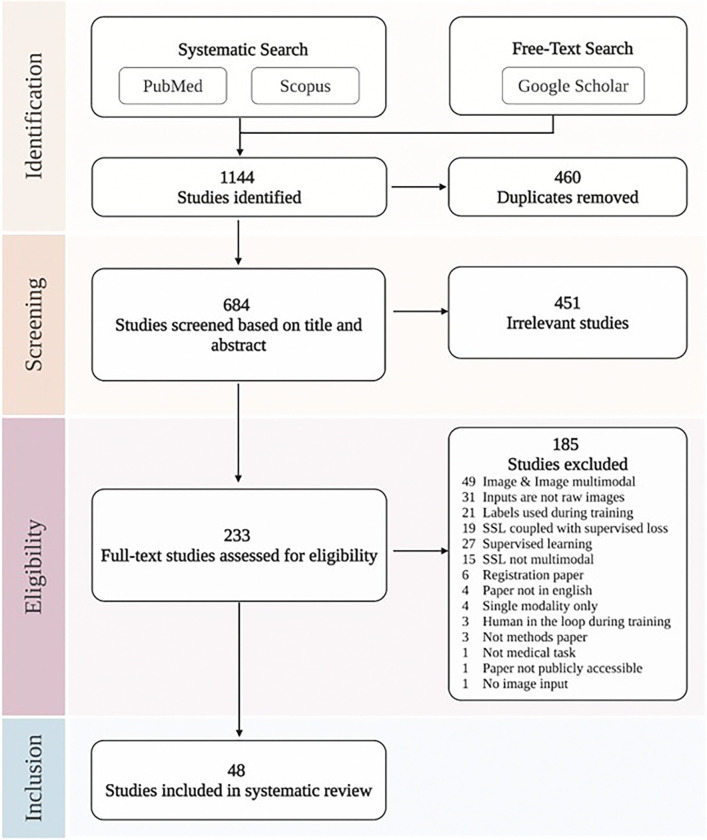
PRISMA flowchart of the study selection process. This figure illustrates the performed identification, abstract screening, and eligibility for inclusion according to the PRISMA guidelines.

**Figure 5:**
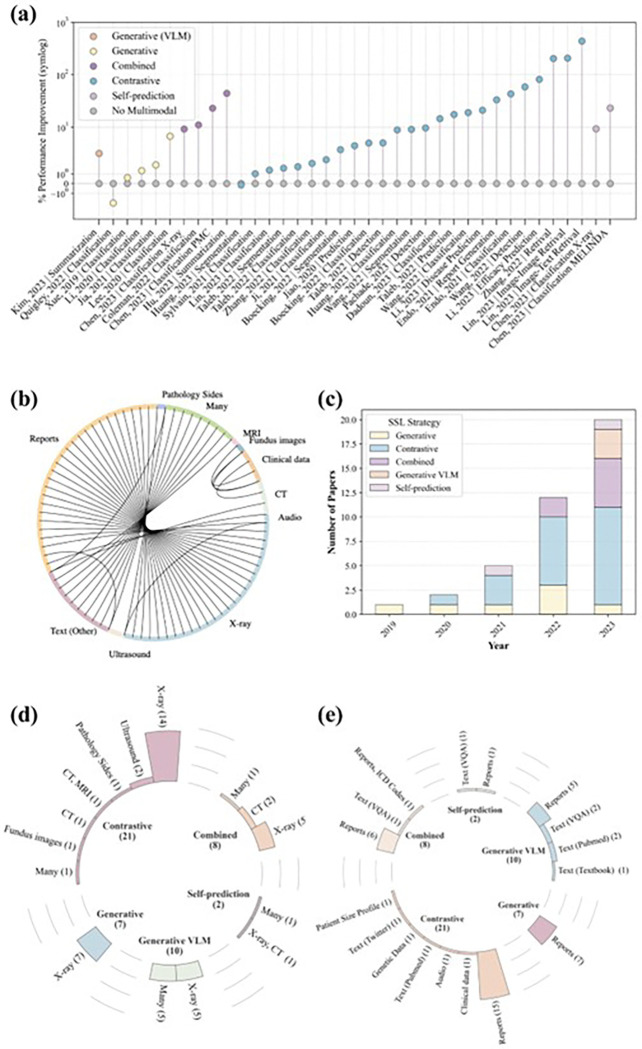
Summary of extracted data from studies included in our systematic review. (**a**) Percentage improvement in downstream task performance using multimodal training compared to single modality approaches. (**b**) Combinations of image and non-image data pairs during SSL pretraining. (**c**) Number of multimodal Foundation Model publications per year categorized by SSL strategy. (**d**) Prevalence of imaging modalities across the SSL pretraining strategies. (**e**) Prevalence of non-imaging modalities across various SSL pretraining strategies.

Our systematic search initially identified 1,144 studies. After removing duplicates and applying our selection criteria to the titles and abstracts (detailed in the Methods section), 233 studies qualified for full-text evaluation. Ultimately, 48 studies met our eligibility requirements and were selected for detailed systematic review and data extraction. Figure 2 illustrates the methods of multimodal SSL pretraining, while Fig. 3 demonstrates various approaches for fine-tuning the pretrained model on downstream tasks. Figure 4 illustrates the study selection and screening process as a flowchart. The extracted data for included studies are listed in Table 1 and Supplementary Data 1. Figure 5 summarizes the data analysis. Supplementary Fig. 1 illustrates how performance improves with increasing dataset size, comparing both multimodal and single-modality approaches.

### 2.1 Terminology and Strategies for Training Multimodal Foundation Models

The development of Foundation Models typically involves a two-stage training process: SSL pretraining and fine-tuning. During the pretraining stage, the vast majority of Foundation Models employ self-supervised strategies – a process that utilizes large volumes of unlabeled or naturally paired data to learn general, transferable, and label-efficient representations. Subsequently, in the fine-tuning stage, the pretrained model is adapted to specific downstream tasks. Owing to the knowledge acquired during SSL pretraining, fine-tuning often necessitates minimal labeled data, and, in some cases, can be accomplished without task-specific labels.

In this section, we provide definitions for different categories of multimodal self-supervised pretraining strategies: contrastive, self-prediction, generative, and generative Vision-Language Models (VLMs) (Fig. 2). Additionally, we illustrate various approaches for adapting pretrained models to downstream tasks through fine-tuning (Fig. 3).

#### Contrastive Learning Models.

Contrastive SSL paradigms presuppose that semantically similar input pairs, termed ‘positive pairs’, should exhibit closer alignment in feature space compared to disparate inputs, or ‘negative pairs’. Pioneering methodologies, exemplified by SimCLR^18^ and MoCo^19^, predominantly focused on unimodal data, specifically images. The core objective underpinning these models is the dual process of minimizing the distance between embeddings of positive pairs and maximizing it between negative pairs. Multiple approaches can be used to form positive and negative pairs, where the most common are various augmentations of the same inputs to constitute semantically similar positive pairs, while augmentations across distinct inputs from negative pairs.

Progressing beyond unimodal frameworks, Contrastive Language-Image Pre-Training (CLIP^20^) integrates contrastive learning across image and textual domains. The key difference to its unimodal predecessors is that CLIP delineates positive pairs as images and their corresponding captions, seeking to co-locate image and textual descriptions within a unified multimodal representation space. This approach has paved the way for further explorations into multimodal contrastive learning, yielding diverse strategies for generating localized positive pairs between images and text, hence discovering more fine-grained image-text associations^21–23^. Notably, the scope of modalities encompassed by recent advancements is not confined to images and text but extends to other modalities, such as acoustic signals, electronic health records, or sensor data, provided the paired modalities convey shared semantic content.

#### Self-prediction Models.

Self-prediction SSL involves the process of masking parts of the input data and subsequently attempting to reconstruct the original, unmodified input (Fig. 2b). Self-prediction first emerged in the natural language processing (NLP) domain, where state-of-the-art models were initially trained through a process called Masked Language Modeling (MLM), which involves predicting the masked words from a sentence^24^. Inspired by this success in NLP, initial experiments in computer vision also adopted this method by obscuring or altering random patches of images and training Convolutional Neural Networks (CNNs) to fill in the gaps as an SSL pretraining method^25,26^. More recently, techniques such as BERT pretraining of Image Transformers (BEiT)^27^ and Masked Autoencoders (MAE)^28^ that utilize self-prediction in conjunction with Vision Transformers (ViT)^29^ have demonstrated superior performance after fine-tuning on various natural image benchmarks compared to their CNN-based predecessors.

In a multimodal setting, one or several of the modalities can be masked out before the reconstruction step^30^. This approach allows the model to leverage the complementary information across multiple modalities when reconstructing the masked segments, thereby facilitating an enhanced understanding of the complex associations between the modalities. Often, corresponding text and images are used, such as X-rays and radiology reports, where e.g., parts of the image or text are masked out, and the information from both modalities is used concurrently to reconstruct the input^31^. However, self-prediction may be extended to any other modalities, such as genetics, blood panels, sensor data, or other medical data.

#### Generative Models.

Generative models have been developed to either reconstruct original inputs or generate new, synthetic data, thereby learning the distribution of training data. Unlike self-prediction SSL methods that focus solely on masking parts of the input and use the rest of the unmodified input to guide the reconstruction process, generative SSL methods aim to reconstruct it as a whole. Hence, while self-prediction can only fill in removed information, generative approaches can generate new data.

Pioneering work on generative models utilized autoencoders^32^. Here, an encoder transforms high-dimensional inputs into a lower-dimensional compressed version (latent representation), followed by a decoder that uses the latent representation to reconstruct the original high-dimensional input. In a multimodal setting, an encoder would take one modality as input, and the decoder would generate another modality^33^ (Fig. 2c). For instance, an encoder can take in medical images as inputs, and the decoder’s task is to generate the corresponding reports^34,35^.

Following autoencoders, Generative Adversarial Networks (GANs)^36^ and diffusion models^37^ have achieved notable success and popularity, particularly for image generation tasks. GANs use a generative model to generate a high-quality output, followed by a discriminator network that tries to distinguish whether the generated output stems from the original data distribution or is a synthetic output. Diffusion models are trained by progressively adding small amounts of artificial noise to an input, with the goal of learning to reverse this process and iteratively denoise the input until it becomes noise-free. Successively adding even small amounts of noise to an input eventually fully converts it to noise. During inference, the model can generate new images that resembles the original data distribution by progressively denoising random noise.

In multimodal settings, both GANs and diffusion models can incorporate other modalities to condition the generation process. A popular approach is using text prompts to guide the generation. For instance, instead of generating random medical images, these models can be prompted to generate specific types of medical images (e.g., chest X-rays with particular abnormalities) by incorporating text embeddings from clinical descriptions into the generation process.

#### Generative Vision Language Models (VLMs).

More recently, a new type of multimodal generative model has emerged as a popular approach to train Foundation Models^38^. Here, the encoder takes in an image and an instruction text prompt, and the decoder generates a desired output, such as a summary or a detailed description of part of the image (Fig. 2d)^11,39^. Typically, this type of generative model can leverage pretrained large language models (LLMs) for both text encoding and decoding, which already possess rich semantic understanding, enabling an intuitive input and output language interface for the user. While the majority of these types of Generative VLMs utilize only text and images, these models may also incorporate other modalities such as genetic data, wearable sensors, and other medical data. Combined SSL Pretraining Approaches. While we have distilled the most common multimodal SSL pretraining approaches into distinct major categories above, many recent studies combine multiple SSL pretraining approaches to potentially enrich the model’s capabilities by allowing it to leverage the benefits of each approach. The combination of multiple approaches is often achieved by directly optimizing the loss functions of each SSL pretraining strategy or weighting the sum of each of the losses. Several studies have shown that this amalgamation has been empirically demonstrated to enhance performance across various downstream tasks, surpassing models pretrained with a singular SSL pretraining strategy.

An illustrative example of such a combined approach is the Contrastive Captioner (CoCa) model^40^. CoCa integrates two SSL pretraining strategies: generative pretraining, where the model learns to generate text descriptions (captions) for given images using a VLM decoder, and contrastive learning, where it learns to match images with their corresponding text descriptions using CLIP. By combining these strategies, CoCa learns both to describe images in detail and to understand the relationship between images and text at a broader level. This dual approach allows the model to develop a more comprehensive understanding of the connection between visual and textual information.

#### Adapting to Downstream Tasks (Fine-tuning).

Following the label-free SSL pretraining approaches described above, models are typically adapted to specific downstream tasks using labeled data (Fig. 3). A common method for doing so involves appending a task-specific head to the pretrained image encoder and fine-tuning the model with a conventional supervised learning regime. This process can be performed in two distinct manners: Firstly, by training the entire or parts of the image encoder end-to-end with the task-specific head, as depicted in Fig. 3a; alternatively, by freezing the encoder and utilizing it solely as a feature extractor for the task-specific head, thereby leaving encoder’s weights unchanged (Fig. 3b).

In the absence of labeled training data, models trained with contrastive learning using images and text can be used to perform zero-shot classification – image classification without the need for any additional training data or labels (Fig. 3c). The method poses a class label as text statements, e.g., “a CT scan with ascites present” and “a healthy CT scan,” and both text prompts are then embedded as text embeddings. The proximity of the text embeddings with that of the embedding of the original image is used to decide what prompt best represents the image^20^. More broadly, this approach extends beyond images and can be applied to multimodal contrastive learning across diverse modalities, including patient health record, genetics data, and other biomedical signals.

Alternatively, prompting is a versatile strategy for tasks requiring text generation, such as image captioning, summarization, or question answering. In this approach, VLMs are given a textual input (the prompt) that guides them in generating the desired outputs (Fig. 3d). However, the effectiveness of prompting can vary significantly based on the model’s initial SSL pretraining objectives, potentially yielding outputs that diverge from expectations. Addressing this challenge, “instruction tuning” has emerged as a novel training paradigm. This method involves further training of VLMs by using explicit pairs of instructions and expected answers tailored to specific downstream tasks. Instruction tuning enhances the model’s ability to follow diverse task-specific prompts and generate text outputs more aligned with the intended task^41^. Some studies have also shown that instruction tuning can enable VLMs to perform a wide range of tasks beyond text generation. For instance, the VLM can be used for classification by instruction tuning it to output classification labels as text^42,43^.

### 2.2 Performance, Methodologies, and Modalities of Multimodal Foundation Models in Medical Imaging

#### Contrastive Learning Models.

Contrastive SSL was utilized in 21 out of 48 studies (Table 1). For imaging modalities used in these studies, X-rays were the most prevalent, featured in 14 studies^21–23,34,44–53^. Ultrasound was used in 2 studies^54,55^, CT images in 1 study^56^, and a combination of CT and MRI in another^57^. Additionally, fundus images^58^, pathology slides^59^, and medical images from PubMed papers^60^ were each employed in 1 study. For the corresponding non-imaging modalities, radiology reports were the most common, appearing in 15 studies^21–23,34,44–53,55^, while 2 studies used other text sources, specifically PubMed image captions^60^ and text from Twitter posts^59^. Genetic data^58^, patient size profiles^56^, clinical data, and speech^54^ were each used once in separate studies.

Eight studies used traditional image-text contrastive learning similar to CLIP^34,44,48,49,51,55,59,60^, 7 studies employed both global and local contrastive learning^21–23,45–47,50^, 1 study adopted a strategy akin to SimSiam^57^, 1 study explored local, global and temporal correspondence^52^ and 4 studies developed novel strategies^53,54,56,58^. The reported average improvement of multimodality over single modality, where available, was: 0.050 AUROC (10 studies^21–23,45,46,49,51,53,54,58^), 0.201 accuracy (1 study^57^), 0.362 Precision@5 (2 studies^49,53^), 0.023 BLEU-2 (1 study^44^), 0.103 F1-score (2 studies^44,55^), 0.028 Dice (3 studies^46,52,58^), and 0.092 mAP (1 study^46^)

While most studies apply contrastive learning between text and images, two studies applied contrastive learning to other combinations of modalities. Taleb et al. was the only study across all categories that combined images and genetic data^58^. They utilized fundus images in conjunction with Single Nucleotide Polymorphisms (SNP) and Polygenic Risk Scores from the UK Biobank to create positive pairs within each patient, using other patients as negative pairs. Their findings demonstrated that this method can enhance fundus pathology detection and facilitate the identification of genetic associations with fundus diseases. Jiao et al. utilized paired ultrasound images and audio of a clinician describing findings during the ultrasound^54^. They created positive pairs between speech and ultrasound at the same time points, while later time points served as negative pairs and audio sections with background noise were used as hard negatives.

Two studies chose non-traditional approaches for collecting paired images and text^59,60^. Rather than using medical images and radiology reports, Zhang et. al. scraped PubMed for papers with medical images and their corresponding captions, yielding a dataset of over 15 million image-caption pairs^60^. The authors used CLIP-style training to train BiomedCLIP, which outcompeted several benchmarks in VQA, image classification and retrieval. Similarly, instead of relying on publicly released datasets or proprietary hospital data, Huang et. al. used Twitter posts of pathology slices with their corresponding text to curate a public dataset^59^.

### None of the studies employed human validation.

#### Self-prediction Models.

Two of the 48 studies utilized self-prediction as an SSL pretraining method (Table 1). Khare et al. presented MMBERT^31^, which used masked language modeling to train a multimodal encoder by randomly masking out words in the image caption and restoring the original caption, jointly utilizing both language and image features. They did not report single modality performance^31^. Chen et al. employed cross-attention between encoders in masked language modeling and masked image modeling to restore both text and images^61^. They demonstrated their methods on X-rays and CT scans, using radiology reports as their second modality. Their work reported an increase of 0.147 in accuracy and 0.075 in AUROC for multimodal over single-modality.

### None of the studies employed human validation.

#### Generative Models.

Generative SSL pretraining was used in 7 out of 48 studies (Table 1). All generative papers used X-ray images as their imaging modality and radiology reports for their corresponding modality. Four out of the 7 papers pretrained their models based on generating the findings section for radiology reports^35,62–64^. The reported average improvement of multimodality over single-modality was 0.002 AUROC (3 studies^35,62,64^) and 0.053 F1 score (1 study^63^). Notably, Quigley et al. reported a higher AUROC for their unimodal text-based model when using 100% of the training data for fine-tuning, but a higher AUROC for their multimodal approach when only a subset of the training data were used.

The remaining 3 studies pretrained their models by generating synthetic chest X-rays based on radiology reports or text prompts^65–67^. Chambon et al. demonstrated a successful adaptation of a general domain image generation model, Stable Diffusion, to generate synthetic chest X-ray images^66^. Chambon et al. further validated the utility of synthetic images by showing a 5% points improvement in classifier performance when trained jointly on synthetic and real images^65^. BiomedJourney showcased the capability to edit chest X-ray images using natural language instructions, enabling the creation of counterfactual images^67^. For instance, the model can generate specific abnormalities on a healthy patient’s chest X-ray using prompts, effectively transforming normal images into ones displaying the requested pathologies.

Two of the 7 studies included human evaluation of the model outputs^65,66^. Chambon et al.^65^ had radiologists evaluate generated X-rays for realism and prompt coherence, finding that while RoentGen demonstrated strong alignment with input prompts, radiologists could still identify the images as synthetic. In another study, Chambon et al. assessed the clinical utility of these synthetic X-rays, determining that diagnostic features were well-preserved in the generated images^66^.

#### Generative VLM.

Ten out of 48 studies^68–77^ leveraged existing text-based Foundation Models (LLMs) to develop VLMs (Table 1). Out of the 10 studies, 5 studies specifically focused on X-ray as their imaging modality^68,71,73,76,77^, while the remaining 5 were capable of analyzing several different imaging modalities. In terms of the corresponding modality, 3 studies used the corresponding radiology reports^68,71,73,76,77^ while 2 used questions from VQA datasets^72,74^. Three studies found creative ways to source corresponding text, including PubMed image captions^69^ and text from publications and medical textbooks^70,75^. Of these studies, 1 reported an improvement of 1.45 ROUGE from multimodal training over single modality training^68^. The remaining studies did not perform this comparison.

Some notable work includes Med-PaLM Multimodal^74^, which emerged as a model capable of encoding and interpreting a wide array of biomedical data, including clinical language, imaging, and genomics, all with the same set of model weights. Med-Gemini^72^ further improves upon Med-PaLM by leveraging the multimodal capabilities of Gemini^12^. In addition, Med-Gemini incorporated self-training and web search integration, enhancing the model’s ability to verify its outputs and improve reliability. MedVersa^75^ introduced an innovative approach using an LLM-powered orchestrator to determine whether to process inputs using the language model alone or integrate specialized visual modeling modules for tasks such as detection, segmentation, and classification, demonstrating improved efficiency and accuracy in medical image analysis.

Several studies demonstrated that generative VLMs need not be proprietary; instead, smaller, open-source VLMs can rival the performance of their larger, closed-source counterparts^69,71,73,76–78^. LLaVA-Med^69^, demonstrated that the open-sourced model, LLaVA^11^, could be successfully adapted to the medical domain in less than a day of training using open-sourced model LLaVA^11^. LLaVA-Rad extends on LLaVA-Med to focus on the task of report generation and further introduced a CheXprompt, a GPT-4-based metric designed to assess the factual accuracy of generated reports. Notably, CheXprompt demonstrated parity with expert radiologist evaluations, showing no statistically significant difference in its assessments. Similar to LLaVA-Rad, MAIRA-2 is a small yet effective report generation model, and demonstrated that generated reports could be grounded by associating generated text with bounding boxes, providing an easier way for physicians to verify the generated report based on visual signals in the image^77^.

Nine of the 10 studies^30,70–77^ employed human validation of the model outputs, where 3 performed human evaluation^70,73,77^, 1 used direct comparison to human performance^75^, 3 used human-driven metrics^69,71,76^, and 2 used human preference and performance evaluation^72,74^. Notably, in side-by-side comparisons, radiologists preferred the AI-generated reports from Med-Gemini^72^ and Med-PaLM-M^74^ approximately 50% of the time. Similarly, Chen et al.^73^ demonstrated that CheXagent significantly increased radiologists’ efficiency, reducing resident reporting time by 36% and improving perceived efficiency in 81% of cases. Bannur et al.^77^ showed that MAIRA-2 produced draft reports requiring minimal corrections, with 91% of generated sentences being acceptable as-is, suggesting potential efficiency gains for radiologists.

#### Combined Approaches.

A combined SSL approach was employed in 8 out of the 48 studies (Table 1). Among these studies, 5 used X-rays as the imaging modality^79–83^, 2 used CT scans^84,85^, and 1 used multiple types of radiology images^86^. For non-imaging modality, 6 studies used radiology reports^79–84^, while 1 study used text from VQA^86^, and 1 used both report and ICD codes^85^. Contrastive learning emerged as the most frequent method in a combined SSL pretraining strategy, being utilized in 6 out of the 8 studies^79–81,84–86^. In 3 of these 6 studies^79,84,86^, contrastive learning was combined with masked modeling, while 2 in the remaining studies^80,81^ combined it with a generative task, and 1 pretrained the model with a pretext task^87^ before contrastive learning^85^. One study employed masked language modeling and image-report matching together^83^, while another study utilized a diverse set of approaches, including Masked Language Modeling, Masked Feature Regression, and Image to Text Matching^82^. Overall, an increase in AUROC of 0.098 (1 studies^82^) and 20.8 in ROUGE-L (1 study^80^) was reported for multimodality over single modality.

Notably, Sangjoon Park et al. trained a model to detect and correct critical errors in radiology reports by leveraging CLIP-style contrastive learning, multimodal masked modeling, and momentum updating of a teacher model akin to DINO training^84^. Pengfei Li et al. combined contrastive learning, masked language modeling, and image text matching to pretrain on multiple large open medical image datasets, followed by fine-tuning on VQA-RAD, PathVQA and SLAKE where their method exceeded state-of-the-art on VQA^86^. Lastly, Blankemeier et al. used a creative approach to train a CT Foundation Model, Merlin, by first utilizing a pretext task^87^ of predicting ICD codes from CT scans and subsequently continuing the training of the model with a CLIP-style contrastive objective between CTs and reports, allowing their model to achieve state-of-the-art performance on numerous tasks^85^. Importantly, this study stands out as one of the few that focuses on ingesting and processing full CT scans, expanding the application of Foundation Models beyond the more commonly studied 2D modalities such as X-rays and addressing the unique challenges and opportunities presented by three-dimensional imaging data.

One study performed human evaluation of the outputs^80^ and one did direct comparison with human performance^84^. Hu et al. conducted a direct human evaluation of AI-generated impressions from X-rays, assessing their readability, accuracy, and completeness, finding that most impressions generated by their model were at least as good as the reference impressions. Park et al. compared zero-shot AI performance against radiologists in identifying clinically significant abnormalities on X-rays, demonstrating that the AI outperformed radiologists on this task.

## 3. Discussion

The purpose of this systematic review is to synthesize the current state of multimodal Foundation Models for medical imaging applications. We propose a unified terminology for multimodal self-supervised strategies commonly used to pretrain Foundation Models. We screened a total of 1,144 papers retrieved based on our search strings and extracted data from 48 included studies. Based on our review, we found that since 2021, contrastive-based methods have prevailed, with combined approaches rising in 2022 and VLM gaining traction in 2023 (Fig. 5c). Additionally, we found that multimodal SSL pretraining generally improved downstream task performance compared to single-modality SSL pretraining, with gains ranging up to 439% across studies (Fig. 5a). The nascent nature of the multimodal deep learning field and the heterogeneity in experimental setups currently precludes definitive conclusions about the superiority of specific multimodal SSL strategies across all medical imaging domains and modalities. Despite these limitations, our findings demonstrate that multimodal Foundation Models offer significant advantages in label efficiency and generalizability across diverse downstream tasks. Furthermore, by integrating signals from multiple modalities, much like physicians do in practice, these models have the potential to facilitate more comprehensive and accurate diagnoses. Therefore, we encourage future research to further explore and refine these approaches, as they hold promise for advancing medical imaging applications and improving patient outcomes.

Our systematic review reveals an emerging trend towards generative VLMs that leverage the advanced capabilities of LLMs for medical tasks. These large models, typically many billions of parameters, power advanced chatbots like ChatGPT and Gemini and demonstrate extensive versatility in handling diverse modalities and performing a wide array of downstream tasks. For instance, Med-PaLM multimodal^74^ showcases the ability to process and interpret biomedical data spanning clinical text, imaging, and genomics, all within a unified model architecture. The trend towards these comprehensive generative models is not only driven by their performance and generalizability but also by their natural language interactive interfaces. These chat-like features can potentially enable nuanced physician-AI collaboration and discussion for diagnosis, moving beyond simple reliance on AI outputs to potentially improve patient outcomes.

However, despite this promise, the sheer scale and computational demands of these generative models can preclude their deployment within hospital firewalls, raising legitimate concerns about the privacy and security of patient health records when transmitted to external model providers. Addressing this challenge, Foundation Models such as LLaVA-Rad^71^ and CheXagent^73^, which are smaller and open-source, rival the capabilities of their larger counterparts, making local deployment more feasible. In addition, adopting federated learning^101–103^ or homomorphic encryption^104,105^ can mitigate privacy risks when sharing data, while knowledge distillation^106–108^ and quantization^109^ offer practical avenues to reduce computational requirements without significantly compromising performance. Furthermore, advances in Foundation Model development come with inherent challenges that must be addressed, such as their tendency to hallucinate, which could lead to potentially harmful misdiagnosis in healthcare settings. The approach taken by models like Med-Gemini^72^, which incorporates online search capabilities to verify its outputs, sets a valuable precedent for enhancing the reliability and safety of AI-assisted medical decision-making. These considerations underscore the importance of balancing model capability, deployability, and safety as we continue to develop and refine Foundation Models for healthcare applications.

Underpinning the development and capabilities of these large Foundation Models is the requirement for vast amounts of unlabeled data. This substantial data volume and diverse dataset not only contribute to the model’s emerging properties but have also been shown to improve the model’s resilience to distribution shift^110^. Such robustness is crucial for deployment in hospital settings, where variations in imaging equipment or patient populations can lead to significant distributional changes. However, patient privacy concerns often restrict access to large-scale medical data, creating a significant hurdle in the development process. The largest publicly accessible medical datasets^92,111–113^ pale in comparison to the internet-scale data used to train general domain Foundation Models. In response to this challenge, several studies in our review have identified innovative approaches to data sourcing, such as leveraging PubMed images and captions^60^, extracting interleaved text and images from medical textbooks^70^, and even mining relevant posts from social media platforms like Twitter^59^. As we move forward in developing more powerful and generalizable medical AI models, these innovative data collection and pairing techniques will likely play an increasingly crucial role in overcoming limitations posed by data scarcity and privacy concerns. However, developers must also be aware that while large-scale datasets from public sources can provide valuable training data, they may not always meet the necessary standards for clinical applications. Therefore, future model development must carefully navigate the tradeoff between data quantity and quality, balancing the benefits of large-scale datasets with the need for high-quality, clinically relevant information to ensure both powerful and reliable AI models for real-world medical applications.

Beyond the challenges of data quantity and sourcing, the types of data integrated are critical for clinical relevance. While many of the papers in our review focused on developing multimodal Foundation Models using medical images and text (Fig. 5b), it is crucial to emphasize that these models should expand beyond natural languages to better align with the multifaceted nature of healthcare. Our review identified several studies that incorporated unique modalities during SSL pretraining, including genetic data^58^, clinical data^57^, ICD codes^85^, speech^54^, and patient size profiles^56^. The inclusion of these diverse modalities provides the model with a more comprehensive view of the patient, mirroring the approach taken by physicians in clinical practice. This multimodal approach not only enhances the model’s diagnostic and prognostic capabilities but also opens up new avenues for discovering complex associations between different modalities. For instance, the ability to correlate genetic data with pathology slides could uncover intricate relationships that might be challenging for human experts to identify independently. Such capabilities have the potential to significantly expand research opportunities in fields such as genetics, pharmacology, and therapeutics. As the field of medical AI continues to advance, it is imperative to develop truly comprehensive multimodal models that can integrate and analyze the full spectrum of patient data available in modern healthcare settings.

Regardless of the specific modalities integrated, assessing the real-world value of these complex models requires careful evaluation. Standard quantitative metrics may not capture clinical nuances, leading some studies to adopt human-centered metrics. In total, we identified 13 studies^65,66,69–77,80,84^ that used human-centered metrics to evaluate their models’ capabilities (see definitions in Data Extraction), where 6 studies employed human evaluation^65,66,70,73,77,80^, 3 studies used human-driven metrics^69,71,76^, 2 studies made direct comparisons to human performance^75,84^, and 2 studies used a combination of human evaluation and performance comparison^72,74^. All 13 studies were within radiology, where 11 focused on radiology report generation^65,66,69–77,80,84^ and 2 focused on X-ray generation^65,66^. The use of human-centered metrics in these studies was likely driven by the challenges of evaluating the clinical utility of generated reports and synthetic X-rays through simple metrics, where human-centered approaches better capture clinical relevance, workflow integration, and expert reasoning. Studies that utilize human preference and performance metrics reveal where AI systems excel in certain analytical aspects while underperforming in others – insights that traditional metrics alone would miss. As these systems move from research to clinical practice, evaluation frameworks must evolve to prioritize clinical impact over isolated technical performance. Ideally, this should be achieved through human-centered metrics that integrate both quantitative and qualitative assessments from diverse human evaluators.

Although robust evaluation is crucial across all applications, we note that a significant proportion of the studies in our review focus on radiology, largely due to the availability of paired radiology images and reports, as well as well-curated and widely accessible public datasets. Although this emphasis has thus far shaped the field, we now observe a growing interest in extending multimodal Foundation Models to other clinical domains, including ophthalmology^114–117^ and pathology^118–121^, trends emerging since the completion of our literature search. We encourage researchers to capitalize on the insights gleaned from radiology-focused efforts and apply these lessons across a broader range of medical specialties, paving the way for truly comprehensive, data-driven care.

### Guideline Overview.

While our systematic review underscores the significant advancements and immense potential of multimodal Foundation Models, realizing this potential in routine clinical practice requires navigating substantial challenges related to data, evaluation, deployment, and clinical integration. The gap between technological capability and practical implementation highlights the need for clear, actionable strategies. Bridging this divide requires a collaborative effort among all stakeholders, model developers, clinicians, policymakers, and dataset curators, to address key challenges hindering clinical adoption, such as potential biases, demonstrating clinical utility, and overcoming practical implementation barriers. Recognizing the critical importance of this interdisciplinary collaboration, we synthesize our findings and observations and provide targeted guidelines for each of those key stakeholders below. The recommendations aim to foster a cohesive approach to developing AI systems that are not only technically impactful but also clinically relevant, ethically sound, and practically implementable in real-world healthcare settings.

### Guidelines for Model Developers.

Model developers should leverage the recent advances in multimodal SSL techniques from the general domain when building medical imaging AI models. However, it is crucial to consider the unique properties and differences between general domain images and medical images when applying these methods^87^. One key difference is that, unlike natural images, where class-defining features often occupy a significant portion of the image, medical images typically have more localized and subtle class-defining features. Consequently, popular multimodal self-supervised methods like CLIP^20^, which rely on learning joint representations between global image and text features, may have limitations in capturing these subtle, localized features in medical images. To address this challenge, innovative approaches have been proposed to adapt methods from the general domain to the specific characteristics of medical images. For example, GLoRIA^23^, ViLLA^88^, and BioViL^89^ demonstrate a promising approach to modifying SSL techniques to better suit the unique properties of medical imaging data. Future developers should also consider introducing technical innovations to adapt general domain methods to meet the specific challenges and characteristics of medical images.

In addition to developing medical imaging-specific methods, developers should also consider using evaluation metrics tailored to specific medical tasks. For instance, in radiology report generation, two common types of metrics are used: (1) lexical similarity-based metrics (i.e. BLUE^90^, ROUGE^91^), which assess whether the model’s outputs are contextually and stylistically aligned with human-written reports, and (2) factual correctness metrics (i.e. F1-CheXpert^92^, F1-RadGraph^93,94^), which evaluate the extent to which the generated reports accurately reflect the imaging findings. While both coherence and factual accuracy are essential for high-quality radiology reports, studies have found that these metrics have limited correlation with manual error scoring performed by radiologists^95^. To address this discrepancy, researchers have proposed novel approaches to evaluate radiology report generation models automatically. For example, LLaVA-Rad^71^ introduced a method that uses GPT-4 to analyze the error types in the generated reports automatically. The resulting metric, CheXprompt, has been shown to have no statistically significant difference compared to human radiologist evaluations, suggesting that it could be used as a substitute for manual radiologist assessment when evaluating the clinical utility of these models. Furthermore, methods like GREEN^96^ have shown that GPT-4’s knowledge can be distilled into smaller, open-source models for report evaluation, eliminating the need for API calls and enhancing accessibility and efficiency for researchers and developers.

Moving forward, future work should prioritize the development and adoption of metrics that are more closely aligned with clinical relevance and utility. However, even more crucial is the need for real-world validation studies, which offer a genuine assessment of how these models perform in practice and ultimately bridge the gap between theoretical promise and tangible impact on patient outcomes. Close collaborations with healthcare providers are essential to pilot these multimodal Foundation Models in diverse clinical settings, ensuring they meet practical clinical requirements and effectively translate into routine patient care.

### Guidelines for Clinicians.

Building clinically useful medical AI models is not solely the responsibility of model developers; clinicians play a crucial role and should actively collaborate with model developers^4^. Often, models are developed based on the availability of datasets rather than addressing a genuine clinical need. Consequently, even if these models achieve high evaluation metrics, their utility may be limited in the absence of a clear clinical application. To ensure the development of clinically relevant AI models, it is essential for clinicians to identify true needs in healthcare settings that can be fulfilled or enhanced by AI. Once a clinical need is identified, clinicians should also identify the modalities that are required to complete the task. Lastly, physicians should determine a specific “action” to pair with the machine learning model’s output to address this need effectively. By defining a “decision-action” pair^97^, AI developers can evaluate the model’s utility based on the estimated net benefit in the context of the clinical need. Identifying a genuine clinical need, the modalities required to address this need, and defining an appropriate decision-action pair are instrumental in creating useful and deployable medical AI models, underscoring the importance of clinician involvement throughout the entire AI model development and deployment process.

Once AI models are deployed in clinical settings, it is imperative for clinicians and healthcare providers to maintain a critical and vigilant approach to their utilization. While these Foundation Models demonstrate impressive capabilities and can operate autonomously, there remains the possibility of errors, underscoring the need for careful oversight to ensure patient safety and reliable decision-making. Clinicians should remain acutely aware of the models’ limitations, including their propensity for hallucination and susceptibility to performance degradation due to distribution shifts. It is crucial for healthcare professionals to monitor the models’ outputs actively, identifying and documenting any errors or inconsistencies observed during clinical use. Establishing a robust feedback loop between clinicians and model developers is essential for the continuous improvement and refinement of these AI systems.

### Guidelines for Policymakers.

Policymakers play a crucial role in shaping the development and deployment of medical imaging AI, particularly in the context of multimodal Foundation Models. To enable responsible innovation while ensuring patient safety, policy interventions should focus on several key areas. Firstly, policymakers should consider establishing an expedited approval pathway for approved multimodal Foundation Models when adapting to unapproved clinical tasks, similar to the FDA’s 510(k) process, to facilitate efficient deployment while maintaining stringent safety standards. This approach is warranted by the demonstrated capacity of Foundation Models to generalize to novel tasks with minimal additional training data. The approval process should distinguish between models utilizing previously approved modalities for inference and those incorporating entirely new modalities, with the latter necessitating a more comprehensive evaluation.

Secondly, policies should mandate sensitivity analyses on combinations of input modalities to assess how these models perform when certain modalities are unavailable, ensuring robustness in real-world clinical scenarios. This requirement is critical as not all modalities may be present in real-world clinical scenarios and model behavior may fluctuate based on available input modalities. For example, a newly admitted patient might not have access to certain medical imaging modalities or clinical data types that the model was trained to rely on. Understanding model behavior in these cases is crucial to prevent unexpected or potentially harmful predictions.

Lastly, as generative AI models become increasingly prevalent in medical imaging applications, policymakers should develop comprehensive evaluation guidelines for generative tasks, such as clinical report generation or summarization. As mentioned in the “Guidelines for Model Developers” section, traditional lexical similarity or factual correctness NLP metrics may be inadequate for evaluating generated medical text. This could involve establishing standards for human expert evaluation of generated reports or incorporating AI-assisted judgment systems, as demonstrated to be feasible in studies like LLaVA-Rad^71^. A structured evaluation framework incorporating both human expert review and AI-assisted assessment, with a mechanism for resolving discrepancies, could enhance the reliability, clinical relevance, and feasibility of these assessments. By addressing these areas, policymakers can foster an environment that promotes the responsible development and implementation of advanced AI models in medical imaging, ultimately leading to improved patient care and outcomes.

### Guidelines for Dataset Curators.

Many existing publicly available datasets on medical images, radiology reports, and other clinical data, are sourced primarily from developed countries, which can lead to biases that disproportionately affect model performance when deployed in developing countries or among minority groups in developed regions, where data representation may be inadequate^98–100^. Additionally, variations in, e.g., medical imaging equipment and protocols across different healthcare settings can introduce distribution shifts, further impacting model generalizability. To mitigate these biases, dataset curators should prioritize diversity in patient demographics, imaging equipment, and clinical protocols, accounting for variations in healthcare infrastructure. Additionally, they should include metadata – such as patient demographics, imaging devices, and scanning protocols – to enable model developers to assess fairness and generalizability across diverse subgroups and clinical settings. By taking these steps to curate diverse and inclusive datasets, we can help ensure that medical AI models are trained and evaluated on representative data and can provide equitable benefits to patients across different regions and demographics.

In conclusion, our systematic review highlights the significant potential of multimodal Foundation Models in advancing medical imaging and healthcare AI. These models demonstrate promising improvements in performance and generalizability across various medical tasks. However, their development and deployment face challenges, including the need for representative and diverse data, privacy concerns, and the need for interpretability and safety in clinical settings. Moving forward, we advocate future research to focuses on (1) developing smaller, privacy-preserving, and deployable models without compromising performance; (2) innovative data collection strategies that respect patient privacy and ensure representativeness across diverse populations; (3) incorporation of diverse non-imaging modalities to better reflect the complexity of healthcare; and (4) rigorous evaluation of these models on clinically relevant downstream tasks using human-centered metrics. As the field progresses, we anticipate that multimodal Foundation Models will play an increasingly crucial role in healthcare, potentially revolutionizing diagnosis, treatment planning, and patient care. However, their successful integration into clinical practice will require continued collaboration between AI researchers, healthcare professionals, and policymakers to ensure these powerful tools are developed and used responsibly, effectively, and ethically.

## Methods

This systematic review was conducted based on the PRISMA guidelines.^122^

### Search Strategy

A systematic literature search was conducted using the two literature databases: PubMed and Scopus. To supplement this search and identify additional relevant studies that may not have been captured by database queries, targeted free-text searches were conducted on Google Scholar. The key search terms were based on a combination of three major themes: “self-supervised learning/Foundation Models”, “medical imaging modalities” and “other medical modalities/multimodal” (see Supplementary Table 1). Search terms for medical imaging were broadly defined to include imaging from all medical fields, i.e., radiology images, fundus photography, whole slide imaging, endoscopy, and echocardiography. The search encompassed papers published between January 1st 2012 and January 1st 2024. The start date was considered appropriate due to the rising popularity of deep learning for computer vision since the 2012 ImageNet challenge. The complete search strings are provided in Supplementary Table 2.

We **included** all research papers in English that used multimodal SSL techniques to develop Foundation Models for medical imaging tasks. We **excluded** studies that used non-human medical imaging data (i.e., veterinarian medical images). We also excluded studies that only used different imaging modalities as their multimodal inputs. Studies that rely on derived imaging characteristics, including biomarkers and radiomic features, as opposed to utilizing raw images directly, are also excluded from consideration. Conference abstracts, review articles, letters to the editor, and any submissions not constituting original research were also excluded. Additionally, studies that did not center on medical imaging, did not employ multimodal SSL pretraining were not considered. Papers focusing solely on image registration were also outside the scope of this review.

Furthermore we constrained our inclusion criteria to studies that applied the multimodal SSL pretrained models to a downstream medical image task. In other words, it was not sufficient for the study to have merely developed a multimodal self-supervised pretrained model; the model had to be evaluated on a clinically relevant task using medical images. We defined a clinically relevant task as one that directly relates to a clinical application or has the potential to inform clinical decision-making. For example, the downstream task of classifying the frame number in a temporal sequence of frames from echocardiography was not considered a clinically relevant task, as it does not provide meaningful information to a clinician to improve patient care.

### Study Selection

The Covidence software (www.covidence.org) was used for screening and study selection. After removing duplicates, studies were screened based on title and abstract. Subsequently, full texts were obtained and assessed for inclusion and data extraction. Study selection was performed by two independent researchers (S.-C.H., M.E.K.J.), and disagreements were resolved through discussion. In cases where consensus could not be achieved, a third arbitrating researcher was consulted (A.S.C.).

### Data extraction

For benchmarking the existing **approaches** (Table 1) we extracted the following data from each of the selected articles: (a) first author, (b) year of publication, (c) medical domain, (d) imaging modalities, (e) non-image modalities, (f) SSL pretraining strategy, (g) whether human-centered metrics were used. The human-centered metrics were divided into four categories: (1) Human Evaluation, in which human evaluators assess the outputs of the model, typically by assigning a score or category, (2) Human-Driven Metrics, where new metrics are created to better align with human preferences, (3) Direct Comparisons to Human Performance, where model performance is directly compared to human performance on the same task, (4) Human Preference and Performance Evaluation, where human evaluators not only assess model outputs but also compare them against human outputs to gauge preference and performance. We classified the specific multimodal SSL pretraining strategy based on the definitions in the “Terminology and Strategies for Training Multimodal Foundation Models” section.

We provide in Supplementary Data 1 all data extracted, including full paper title, SSL, medical domain, pretraining dataset, dataset size, image encoder, other modalities encoder, imaging model weight initialization, other modalities model weight initialization, multimodal self-supervised model performance(s), single modality model performance(s) when available, downstream task(s), and evaluation metric(s). We extracted AUROC whenever this metric was reported; otherwise, we prioritized the F1 score over accuracy and sensitivity. For NLP tasks, we prioritized longer subsequences (i.e. ROUGE-2 over ROUGE-1, ROUGE-L over ROUGE-2, etc.). We used ROUGE over BLEU due to its recall-oriented nature, which is crucial for capturing all relevant medical information. When the article contained results from multiple models (i.e., ResNet and Vision Transformer) on the same task, metrics from the experiment with the best-performing model were extracted. When the authors presented results on multiple clinical tasks, we extracted metrics for each of the downstream tasks. In instances where a particular clinical task was evaluated across several datasets, we selected the highest performance from among the datasets. Single-modality baseline performance, model architecture, SSL pretraining dataset, and initialization were extracted when available in the manuscript.

### Limitations of the Review

A notable constraint arises from the inherent publication bias within the extant literature, which predominantly features studies reporting positive outcomes. Such bias may inadvertently lead to an inflated perception of the efficacy associated with multimodal Foundation Models. Our examination was deliberately confined to literature published subsequent to the year 2012, thereby excluding works that predate the advent of deep learning in the realm of computer vision. The heterogeneity presented in the methodologies of the reviewed studies, encompassing diverse imaging modalities, varied performance metrics, and distinct research objectives, precludes a comprehensive quantitative synthesis or direct comparison of the relative benefits conferred by different SSL pretraining strategies. Moreover, the classification of multimodal SSL approaches within each analyzed study was subject to a certain level of subjectivity, especially in instances involving innovative, non-traditional, or hybrid methodologies. Furthermore, the selection criteria for studies were specifically tailored to the domain of medical images. This focus inherently limits the breadth of our review, overlooking the versatility of self-supervised pretrained models, which hold significant promise across a spectrum of other modalities.

## Figures and Tables

**Table 1:** Overview of multimodal self-supervised Foundation Models included in the systematic review. This table shows the included studies along with their medical domain, image modality, non-image modality, SSL pretraining method, and whether human validation was used. All extracted data appears in Supplementary Data 1.

Authors	Year	Medical Domain	Image Modality	Other Modalities	SSL Pretraining Strategy	Human Validation
Jong Hak Moon^83^	2022	Radiology	X-ray	Reports	Combined	No
Matthew Coleman^82^	2022	Radiology	X-ray	Reports	Combined	No
Hong-Yu Zhou^81^	2023	Radiology	X-ray	Reports	Combined	No
Jinpeng Hu^80^	2023	Radiology	X-ray	Reports	Combined	Human Evaluation
Ke Zhang^79^	2023	Radiology	X-ray	Reports	Combined	No
Pengfei Li^86^	2023	Many	Many	VQA	Combined	No
Sangjoon Park^84^	2023	Radiology	CT	Reports	Combined	Direct Comparison to Human Performance
Louis Blankemeier^85^	2024	Radiology	CT	Reports, ICD Codes	Combined	No
Jianbo Jiao^54^	2020	Radiology	Ultrasound	Audio	Contrastive	No
Mark Endo^44^	2021	Radiology	X-ray	Reports	Contrastive	No
Tristan Sylvain^45^	2021	Radiology	X-ray	Reports	Contrastive	No
Zhanghexuan Ji^21^	2021	Radiology	X-ray	Reports	Contrastive	No
Abdullah-Al-Zubaer Imran^56^	2022	Radiology	CT	Patient Size Profile	Contrastive	No
Aiham Taleb^58^	2022	Optomology	Fundus Image	Genetics	Contrastive	No
Benedikt Boecking^22^	2022	Radiology	X-ray	Reports	Contrastive	No
Fuying Wang^46^	2022	Radiology	X-ray	Reports	Contrastive	No
Giorgio Leonardi^47^	2022	Radiology	X-ray	Reports	Contrastive	No
Gongbo Liang^48^	2022	Radiology	X-ray	Reports	Contrastive	No
Yuhao Zhang^49^	2022	Radiology	X-ray	Reports	Contrastive	No
Hind Dadoun^55^	2023	Radiology	Ultrasound	Reports	Contrastive	No
Kangshun Li^57^	2023	Radiology	CT and MRI	Clinical Data	Contrastive	No
Nathan Hadjiyski^50^	2023	Radiology	X-ray	Reports	Contrastive	No
Samiksha Pachade^51^	2023	Radiology	X-ray	Reports	Contrastive	No
Sheng Zhang^60^	*2023*	Many	Many	PubMed Image Captions	Contrastive	No
Shih-Cheng Huang^23^	2023	Radiology	X-ray	Reports	Contrastive	No
Shruthi Bannur^52^	2023	Radiology	X-ray	Reports	Contrastive	No
Xing Wu^34^	2023	Radiology	X-ray	Reports	Contrastive	No
Zhi Huang^59^	2023	Pathology	Pathology Slides	Text from Twitter Posts	Contrastive	No
Zudi Lin^53^	2023	Radiology	X-ray	Reports	Contrastive	No
Yuan Xue^62^	2019	Radiology	X-ray	Reports	Generative	No
Changhwan Lee^63^	2020	Radiology	X-ray	Reports	Generative	No
Xing Jia^35^	2021	Radiology	X-ray	Reports	Generative	No
Keegan Quigley^64^	2022	Radiology	X-ray	Reports	Generative	No
Pierre Chambon^65^	2022	Radiology	X-ray	Reports	Generative	Human Evaluation
Pierre Chambon^66^	2022	Radiology	X-ray	Reports	Generative	Human Evaluation
Yu Gu^67^	2023	Radiology	X-ray	Reports	Generative	No
Gangwoo Kim^68^	2023	Radiology	X-ray	Reports, Prompts	Generative VLM	No
Zhihong Chen^73^	2024	Radiology	X-ray	Reports	Generative VLM	Human Evaluation
Juan Manuel Zambrano Chaves^71^	2024	Radiology	X-ray	Reports	Generative VLM	Human-Driven Metrics
Khaled Saab^72^	2024	Many	Many	VQA	Generative VLM	Human Preference and Performance Evaluation
Tao Tu^72^	2024	Many	Many	VQA	Generative VLM	Human Preference and Performance Evaluation
Chunyuan Li^69^	2023	Many	Many	PubMed Image Captions	Generative VLM	Human-Driven Metrics
Michael Moor^70^	2023	Many	Many	Text From Medical Publications and Books	Generative VLM	Human Evaluation
Hong-Yu Zhou^75^	2024	Many	Many	Medical Text	Generative VLM	Direct Comparison to Human Performance
Shruthi Bannur^77^	2024	Radiology	X-ray	Reports	Generative VLM	Human Evaluation
Stephanie Hyland^76^	2024	Radiology	X-ray	Reports	Generative VLM	Human-Driven Metrics
Yash Khare^31^	2021	Many	Many	VQA	Self-prediction	No
Zhihong Chen^61^	2023	Radiology	CT and X-ray	Reports	Self-prediction	No

The figure illustrates that multimodal SSL represents a small but rapidly growing subset of medical deep learning literature. Publication counts were aggregated using keyword groups (see Supplementary Table 1). For example, “Medical AI” combines the “Deep Learning” and “Medical Imaging” groups, while “Medical AI + Self-supervised Learning” includes the prior two groups plus the “Self-supervised Learning” group. Specific keywords for each group are detailed in the Methodology section and Supplementary Tables 1 and 2. The Y-axis is in log scale.

## Data Availability

All data extracted from the papers and used for analyses in this review are available in Supplementary Data 1. The complete set of keyword groups appears in Supplementary Table 1. The exact search strings used in Scopus and PubMed are provided in Supplementary Table 2. The inclusion and exclusion criteria for screening, along with the detailed data extraction strategy, are described in the Methods section.
